# Balance in Parkinson's disease patients changing the visual input

**DOI:** 10.1590/S1808-86942011000500019

**Published:** 2015-10-22

**Authors:** Hamlet Suarez, Dario Geisinger, Enrique D. Ferreira, Santiago Nogueira, Sofia Arocena, Cecilia San Roman, Alejo Suarez

**Affiliations:** 1 MD. PHD. (Prof. of Otolaryngology.CLAEH School of Medicine. Director of Otoneurology British Hospital. Montevideo); 2 Mr. (Researcher, Laboratorio de Otoneurologia); 3 Eng. PhD. (Aggregate Professor, UCUDAL - Uruguay); 4 Mr. (Researcher, Laboratorio de Otoneurologia); 5 Mrs (Researcher, Laboratorio de Otoneurologia); 6 Mrs (Researcher, Laboratorio de Otoneurologia); 7 MD, MSc (ENT department, British Hospital. Montevideo. Researcher, Laboratorio de Otoneurologia)

**Keywords:** linear models, parkinson disease, postural balance, visual perception

## Abstract

**Abstract:**

The description of the postural responses in Parkinson's disease patients when visual information changes from a stable to a moving visual field analyzing the impact on balance in these patients.

**Methods (Clinical):**

Limits of Stability, Body center of pressure and balance functional reserve were measured by means of the force platform in 24 Parkinson's patients in stages 1 and 2 of the Boher classification and 19 volunteers as a control group. Both groups were stimulated with 1-Static visual field and 2-horizontal optokinetic stimulation using a virtual reality system. Postural responses were analyzed using the inverted pendulum as mathematical model.

**Results:**

While the control group didn't show significant differences on the postural control between the two sensory conditions (COP *p*=0.0017, BFR *p*=0.0025), Parkinson's patients presented significant differences in the area of the center of pressure and the balance functional reserve values between static visual field and optokinetic stimulation. (COP *p*=0.0017, BFR *p*=0.0025).

**Conclusions:**

The results support the hypothesis about the influence of the changes in the visual information in triggering balance control disorders in Parkinson's patients. It is discussed the interest of these fact in the assessment and the rehabilitation programs of this disease.

## INTRODUCTION

Balance disorders in patients with Parkinson's disease (PD) manifest in different stages of the disease, even in the initial stages, thus increasing disability with falls and freezing of gait (FOG). Although balance is typically preserved early in the course of idiopathic PD, many surveys have shown a higher incidence of falls (and their consequences) with rates near 70% in patients in the initials stages of the Boher classification. Fall rates are higher in studies that also include “near falls”^1^.

Recent studies have shown that sensory input and sensory motor processing appear to be relevant in clinical issues in PD, such as instability in open spaces, postural control disorientation, or FOG^2^, ^3^, ^4^, ^5^, ^6^, ^7^, ^8^. One of the interesting points about the relationship between the changes in the cues of sensory information and postural control is the possibility to assess postural responses with these changes in order to understand all of the mechanisms involved in balance disorders in patients with PD. Also, this information could be useful for introducing sensory stimulation in PD rehabilitation protocols in an attempt to achieve postural adaptation.

This study aims to understand the role of changes in one of the most important visual cues (motion) in generating alterations on the postural control model of these patients and analyzing which is the most sensitive parameter for the measurement of altered postural responses.

**Figure 1:**
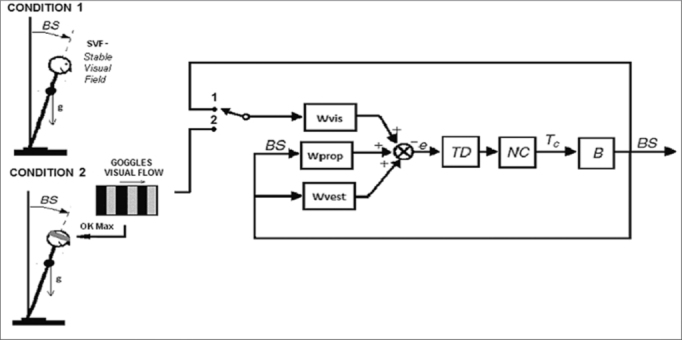
Model of the postural control system - (BS) Body-in-space is the angular desviation from the vertical, (B) human body as an inverted pendulum, (NC) Neuromuscular controller, (TD) Time Delay and Wvis, Wprop, Wvest weighting factors of the visual, proprioceptive and vestibular sensors respectively.

We studied the postural responses in patients with early stage PD when they have a stable visual field (SVF) and when they receive a visual input with a moving field (optokinetic stimulation). The spatial behavior of the body center of pressure (COP) with these two visual input conditions has been analyzed based on the inverted pendulum model (Figure. 1). Figure 1 shows a typical model of the postural control system. This feedback control model is commonly used to describe the mechanism of how humans are able to maintain balance.

In this model the human body (B) is represented as an inverted pendulum with axis through both ankle joints. (NC) is the Neuromuscular Controller that generates the corrective torque (Tc) from the sensory information to stabilize the body. (TD) represents the delay in the transmission, processing, and muscle activation. An internal orientation error signal *e* is formed from a weighted combination of visual, vestibular, and proprioceptive information with weighting factors Wvis, Wvest, and Wprop, respectively. (BS) Body-in-space is the angular deviation from upright stance. In Figure 1 two conditions are shown; Condition 1 represents static visual field (SVF), where the visual input is the angular deviation; Wvis closes the feedback loop with the switch in position 1. Condition 2 represents optokinetic stimulation, where the input is the visual flow presented on the goggles; Wvis closes the feedback loop with the switch in position 2.

The goal of this study is the assessment of the postural control in Parkinson's patients without clinical significant complaint on the balance (Stages 1 and 2 of the Boher classification) when the visual field has a stable frame or a visual input with a constant velocity flow, suggesting a mechanism of alteration on the postural control model with changes in the motion cue in the visual information. We also evaluate the balance functional reserve (BFR) as parameter in the measurement of postural control behavior when these patients receive different sensory stimulation. Functional reserve is a way to describe the range of operation for a specific organ or system, such as renal, respiratory or cardiac. The BFR is a parameter which we have introduced to assess the relationship between the limits of stability (LOS) and the COP area, and quantifies the remaining swaying capacity under different sensory conditions.

## METHODS

### Population

Twenty-four patients in stages 1 and 2 of the Boher classification of PD were studied. (Mean age 66.5 ± 8.5) They were assessed by neurologic bedside examinations, psychological testing using a depression scale, cognitive evaluations, brain MRIs, and SPECT scans of the brain for the measurement of dopaminergic transportation. Patients with musculoskeletal disorders, cognitive impairment, or neuropathy were excluded from this study. All studied patients were being treated with levodopa. Informed consent was obtained from the patients according to the ethical standards of the Helsinki Declaration (1975, revised 1983) and ethical approval was received from the Catholic University of Uruguay (www.ucu.edu.uy/Portals/0/…/Etica/PROTO-aprob-hasta%20DIC09.doc). A control group (CG) of 19 volunteers was recorded under the same conditions as the PD patients (Mean age 62.3 ± 12.7).

### Stimulation Paradigm

Data was obtained using a force platform with a sampling rate of 50 Hz. Subjects performed a posturography session using the BRU (Medicaa System), which includes measurement of the LOS and the COP under a SVF and under visual optokinetic stimulation (60o/s). All subjects where checked for a constant distance between the toes as marked on the force platform (approximately 8 cm) in all exercises.

### Limits of Stability

**Figure 2:**
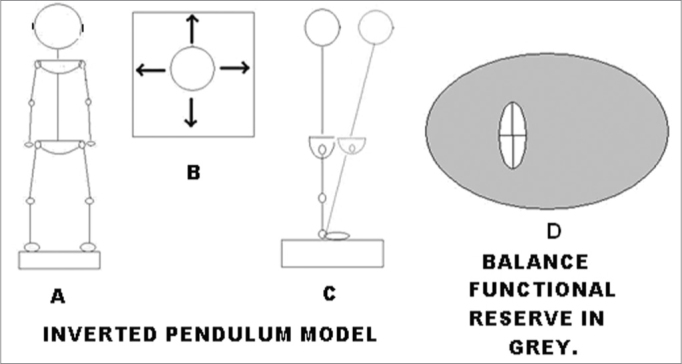
LOS and BFR - Schematic representation of the LOS procedures showing subject in quiet stance (A), direction of the oscillations to test the LOS (B) and the ankle strategies to perform the test (C). The grey area between the LOS and the CE represents the BFR (D).

Instructions to carry on the LOS measurements include swaying forward, backward, and laterally until the maximum was reached without losing balance or lifting the feet. This should be performed using an ankle strategy exclusively from a starting point at which the subject is in a basal position in quiet stance. This generates a cross-like pattern in the COP record. Figure 2A shows the subject in a quiet stance, with the oscillations he ought to perform (top view, Figure 2B) and when swaying in the forward direction (lateral view, Figure 2C). Quantification of the LOS is performed by calculating an ellipse that will estimate its area. This is achieved by approximating the sway pattern to an ellipse, using the maximum and minimum of the total distance swayed in the medio-lateral and anteroposterior axes:

### Center of Pressure (COP)

The COP area is estimated by the ellipse of confidence (CE) for each exercise. The area of the CE at 95% is computed as:

$$\begin{array}{l} a=\frac{\text{max}\left(cop_{x}\right)-\text{min}\left(cop_{x}\right)}{2}\\ b=\frac{\text{max}\left(cop_{x}\right)-\text{min}\left(cop_{y}\right)}{2}\\ A\_LOS=\pi^{*}a^{*}b^{*} \end{array}$$

For the area of the COP during optokinetic stimulation,(60 o/s) we took the maximum value of the COP between the right to left and left to right stimulation (OK).

### Balance Functional Reserve

We determined the ratio of the area of the LOS and the area of the CE giving a measurement on how much area of the LOS is still available for the patient to safely sway. The BFR is presented as the percentage of the total swaying capacity (LOS) still available to sway. Figure 2D graphically shows the meaning of the BFR. The gray area represents the area of the LOS without the CE of an exercise. The BFR quantifies the percentage of the gray area compared to the entire area of the LOS, computed as:

$$BFR\left(\%\right)=\left(1-\frac{Area\_CE}{Area\_LOS}\right)*100$$

### Data Analysis

Statistical analysis was performed with the Wilcoxon test to compare SVF and OK in each population. The Mann -Whitney U test was used to compare between populations for each parameter (both with an alpha at 5%).

To determine the discriminative characteristics of the OK parameter, ROC curves were used. These curves are plotted using the specificity and sensitivity of the sample and determining the cut-off point by maximizing these values. A common value to compare between classifiers is to use the area under the ROC curves (AUC), which is a value between 0.5 (random guessing) and 1 (perfect classifier; 12). All data was processed using Matlab.

## RESULTS

Table 1 shows the LOS, COP and BFR values (With SVF and OK stimulation) for both populations.

**Table 1:** 

Control Group					PD Patients		
	Units	min	mean	max	min	mean	Max
LOS	cm2	57	270	748	31	146	281
COP SVF	cm2	0.91	3.97	11.5	1.2	8.45	43.6
COP OK	cm2	0.68	3.23	11.4	0.85	15.5	80.1
BFR SVF	%	95.88	98.31	99.88	50.53	91.5	99.47
BFR OK	%	91.77	98.35	99.74	31.43	86.76	99.45

Mean, minimum and maximum values for all parameters measured (LOS, COP) and calculated (BFR).

### COP and BFR compared in CG and PD

COP and BFR values in the CG didn't show significant difference between SVF and OK stimulation (COP *p*=0.1531, BFR *p*=0.1161).

**Figure 3:**
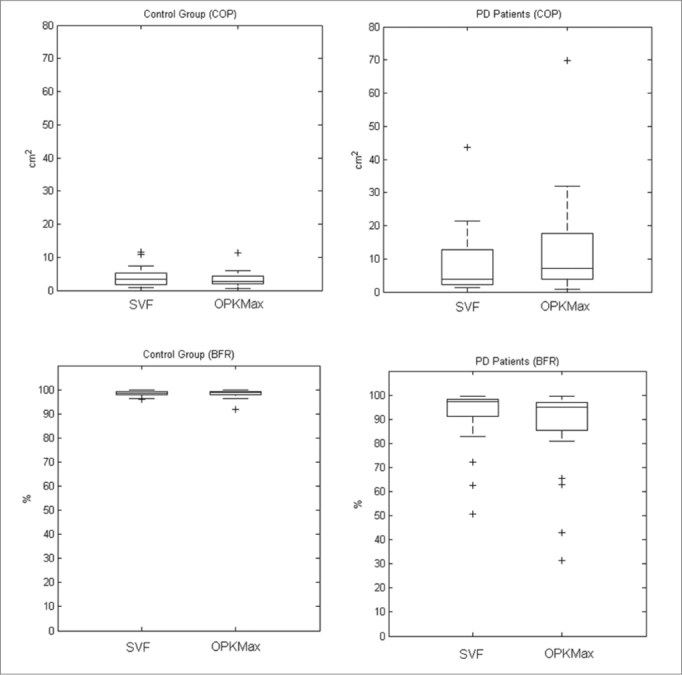
Boxplot comparing results for the COP of the CG between SVF and OK conditions (upper-left), BFR of the CG between both conditions (lower-left), COP of the PD group between both conditions (upper-right) and BFR of the PD group between both conditions (lower-right).

For the PD patients there was significant difference in COP and BFR values between both conditions (COP *p*=0.0017, BFR *p*=0.0025). Figure 3 presents box plot results for SVF and OK conditions for both populations.

### COP and BFR compared between CG and PD in the two sensory Conditions (SVF-OK).

*A comparison between the 2 groups (CG and PD) was done to study the behaviour of the COP and BFR in SVF and OK conditions.*

COP with SVF showed no difference between the two populations (*p*=0.1992). While LOS, BFR with SVF and both COP and BFR for OK stimulation showed a significant difference between CG and PD patients (*p*<0.001, *p*=0.0035, *p*<0.001 and *p*<0.001).

**Figure 4:**
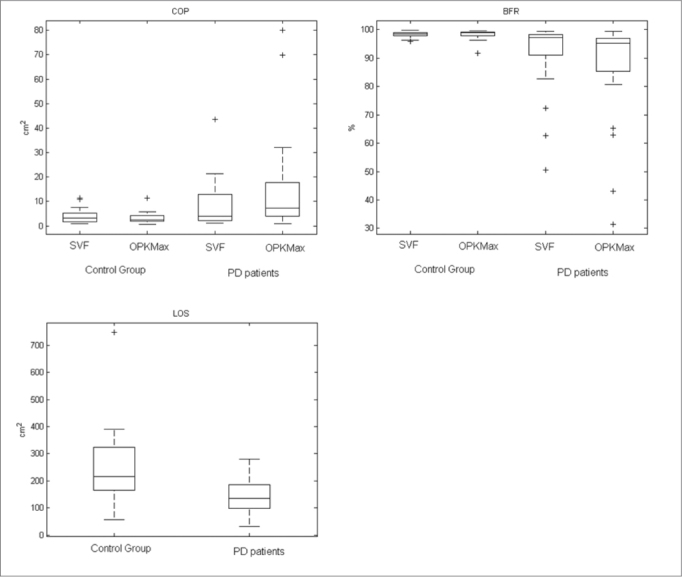
Box plot of the COP of both groups with SVF and OK conditions (Upper left). Box plot of the BFR in the same groups and conditions (Upper right). Box plot of the LOS comparing the CG and PD patients (Lower right).

Figure 4 shows the box plot of the CG and PD patients for all parameters (LOS, COP, BFR).

### ROC analysis

**Figure 5:**
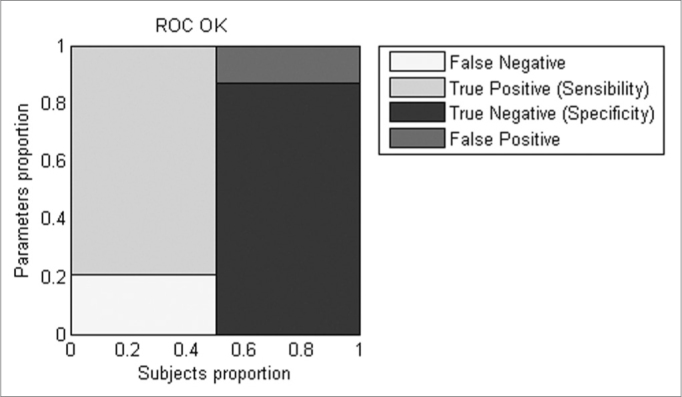
Graphic representation of the sensitivity and specificity calculated by ROC curves for the BFR with OK condition.

BFR with OK stimulation proved to be a better discriminator under ROC analysis than LOS, COP in both conditions and BFR with SVF (AUC BFR OK = 0.89583, AUC LOS = 0.78299, AUC COP SVF = 0.61979, AUC COP OK = 0.81771, AUC BFR SVF = 0.74653). BFR for OK presented a sensitivity of 79.2% and a specificity of 87.5% (Figure 5) better than all other parameters except for the LOS which has lower specificity yet better sensitivity.

## DISCUSSION

Postural control and gait alterations in patients with PD involve sensory input, sensory motor processing, and motor coordination disorders. This study focused on how a change in one visual information (motion) cue modifies and alters postural responses in patients with PD. In addition, the BFR is analyzed as a suitable parameter for measuring these postural responses. The results show that while OK stimulation in the normal subjects (CG) did not produce changes in the COP values compared to a stable visual field, patients with PD had a significant increase in the COP area. The BFR values in this sensory condition are reduced significantly, which suggests that the “safe area” for the body swaying in these patients is less than when they are subjected to a stable visual field, thereby increasing the risk of falls. The results suggest the importance of the visual sensory information on the postural control response in PD patients.

The model of postural control system described in Figure 1 may help to understand the meaning of these findings. Many authors have suggested that the postural control system in humans alters the weighting of sensory orientation cues as environmental conditions change^9^, ^10^, ^11^. The dynamic behavior of the model (Figure. 1) changes according to the values of the three weighting factors. Each factor represents the weight that the central nervous system will give to the information coming from its respective sensor (visual, vestibular or somatosensory). As suggested by Peterka^11^, the values of the weighting factors can be altered according to the environment in which the subject is immersed, specifically discarding information which is not suitable to correctly estimate the deviation from the vertical and prioritizing those inputs which may be giving more accurate information. Our results together with those proposed by Peterka suggest that the control group can minimize the weighting of the visual input when an optokinetic flow is present and thus maintaining similar values of BFR in both conditions. On the other hand, either by minimizing the visual weighting, PD patients must rely on their somatosensory cues which are likely to be impaired^12^ or they do not correctly discard the visual information. This may explain the deterioration in the results when the PD patients are stimulated with a “dynamic visual field” (OK stimulation).

## CONCLUSIONS

This study shows the impact on balance and postural control due to changes in one of the visual cues (motion) in PD patients. Postural control skills in these patients is impaired when they are submitted to a constant visual flow. As a consequence, it shows the risks of instability and falls in open spaces. This paper also suggests the use of BFR to measure postural control with changes on the sensory input.
